# Factors associated with suicide risk in young women with premenstrual dysphoric disorder: a population-based study

**DOI:** 10.47626/2237-6089-2023-0718

**Published:** 2025-09-09

**Authors:** Francine Zanette Machado, Manuela Silva Silveira da Mota, Ester Pereira dos Santos, Divya Prasad, Taiane de Azevedo Cardoso, Benicio N. Frey, Karen Jansen, Luciano Dias de Mattos Souza, Thaise Campos Mondin, Flavio Kapczinski, Fernanda Pedrotti Moreira, Ricardo Azevedo da Silva

**Affiliations:** 1 Universidade Católica de Pelotas Pelotas RS Brazil Programa de Pós-Graduação em Saúde e Comportamento, Universidade Católica de Pelotas, Pelotas, RS, Brazil.; 2 Universidade Federal do Rio Grande do Sul Hospital de Clínicas de Porto Alegre Laboratório de Cronobiologia e Sono Porto Alegre RS Brazil Laboratório de Cronobiologia e Sono, Hospital de Clínicas de Porto Alegre (HCPA), Universidade Federal do Rio Grande do Sul (UFRGS), Porto Alegre, RS, Brazil.; 3 UFRGS Porto Alegre RS Brazil Programa de Pós-Graduação em Psiquiatria e Ciências do Comportamento, UFRGS, Porto Alegre, RS, Brazil.; 4 St Joseph's Healthcare Women's Health Concerns Clinic Hamilton ON Canada Women's Health Concerns Clinic, St Joseph's Healthcare, Hamilton, ON, Canada.; 5 Deakin University School of Medicine Institute for Mental and Physical Health and Clinical Translation Geelong Australia Institute for Mental and Physical Health and Clinical Translation (IMPACT), School of Medicine, Deakin University, Geelong, Australia.; 6 McMaster University Department of Psychiatry and Behavioural Neurosciences Hamilton ON Canada Department of Psychiatry and Behavioural Neurosciences, McMaster University, Hamilton, ON, Canada.; 7 St Joseph's Healthcare Hamilton Hamilton ON Canada Mood Disorders Program, St Joseph's Healthcare Hamilton, Hamilton, ON, Canada.; 8 Universidade Federal de Pelotas Pelotas RS Brazil Pró-Reitoria de Assuntos Estudantis (PRAE), Universidade Federal de Pelotas, Pelotas, RS, Brazil.

**Keywords:** Premenstrual dysphoric disorder, suicidality, youth, women's mental health

## Abstract

**Objective:**

Women with premenstrual dysphoric disorder (PMDD) are more likely to report suicide ideation and behavior when compared to women without PMDD. However, there is a lack of studies investigating the risk factors for suicide risk in women with PMDD. Thus, the aim of this study is to assess the factors associated with suicide risk in young women with PMDD.

**Methods:**

This is a cross-sectional study including 128 young women with PMDD who were recruited from the community. PMDD and suicide risk were assessed by trained psychologists using the Mini International Neuropsychiatric Interview (MINI-PLUS). Suicide risk evaluation includes six questions that assess suicidal intention, planning, and previous attempts. Subjects who answer yes to any of the six questions are classified as having current suicide risk.

**Results:**

The prevalence of current suicide risk in women with PMDD was 28.1%. The factors associated with suicide risk in this population were: presenting current panic disorder (odds ratio [OR]: 18.71 [95% confidence interval {95%CI} 1.02-343.27], p = 0.048), a non-white skin color (OR: 4.18 [95%CI 1.28-13.61], p = 0.018), greater severity of depressive symptoms (OR: 1.22 [95%CI 1.12-1.32], p < 0.001), and history of childhood trauma (OR: 1.04 [95%CI 1.01-1.08], p = 0.010).

**Conclusion:**

Our findings indicate that there are key sociodemographic and clinical factors associated with suicide risk in young women with PMDD, enabling clinicians to identify at-risk individuals who could benefit from further screening and interventions.

## Introduction

In the most recent version of the Diagnostic and Statistical Manual of Mental Disorders, 5th edition, Text Revision (DSM-5-TR), premenstrual dysphoric disorder (PMDD) remains included as a diagnostic category in the depressive disorders section,^1^ where it was included since the DSM-5.^2^ PMDD is defined as a combination of intense changes in behavior, cognition, mood and somatic symptoms that occur during the luteal phase of the menstrual cycle, with at least one of the following symptoms presented: marked affective lability, marked irritability or increase in interpersonal conflicts, marked depressed mood or anxiety.^1^ It is known that women who suffer from PMDD present symptoms that cause substantial clinical suffering or impairment in important areas of their daily lives, such as difficulties in interpersonal relationships.^3^

Several studies have shown an association between PMDD and other co-morbid psychiatric disorders, including depression, social phobia, specific phobia, and bipolar disorder.^4–6^ When there is an association between PMDD and other mood disorders, particularly depression and bipolar disorder, affective symptoms tend to be exacerbated.^7,8^ Furthermore, studies have also shown and association between PMDD and suicidal behavior or ideation.^9–11^ According to a recent meta-analysis, women with PMDD are seven times more likely to present suicide risk when compared to women without the disorder and four times more likely to have suicidal ideation when compared to women without PMDD.^12^ However, to the best of our knowledge, there are no studies investigating the factors associated with suicide risk in young adult women with PMDD.

Our previous report showed that the prevalence of PMDD was 17.6% among young adult women recruited from a community sample. In that study, we found that women with PMDD were twice more likely to present suicide risk as compared to women without PMDD.^5^ Additionally, we recently showed that the incidence of suicide risk was 8.5% in a community sample of young adults.^13^ Importantly, our utilization of machine learning techniques revealed that female sex was one of the most important risk factors for incident suicide risk in our sample.^13^

Considering the high prevalence of suicidal ideation and behavior in young adults and in women with PMDD, it is important to understand which factors are associated with suicide risk in this specific population, so that early detection strategies can be implemented. Thus, the aim of the present study is to assess the factors associated with suicide risk in young adult women with PMDD.

## Methods

### Study design

This is a cross-sectional study nested within a larger cohort study entitled "Psychosocial and Biological Factors in Bipolar Disorder: a population-based cohort of young adults," which took place in the city of Pelotas, state of Rio Grande do Sul (Brazil).

### Setting and participants

In the first wave, the sample was selected by clusters, considering a population of 39,667 young adults with ages ranging from 18 to 24 years, according to the current census of 448 sectors of the city. Aiming to assure the necessary sample size, 89 census-based sectors were randomly selected. The selection of the houses in the sectors was performed according to a systematic sampling, the first one being the house at the corner pre-established by the Brazilian Institute of Geography and Statistics (Instituto Brasileiro de Geografia e Estatística, IBGE) as the beginning of the sector, the interval of selection was determined by skipping two houses. After the identification of the subjects, 1,762 young adults between the ages of 18 and 24 years were approached. Among these, 202 were not found or refused to participate in the study. Thus, the total sample consisted of 1,560 young adults at baseline (880 women, and 680 men), which took place between 2007 and 2009. The present article only includes data from the second wave of data collection, which took place between 2012 and 2014. We only included data from the second wave in this study, because we did not assess PMDD in the first wave of the study. Individuals who participated in the first wave were invited to participate in the second wave. In the second wave, the total sample included 1,244 young adults, and 128 women were diagnosed with PMDD. The inclusion criteria in this study are: (1) having participated in the second wave of the cohort study; (2) being a female; and (3) having a probable diagnosis of current PMDD, according to the Mini International Neuropsychiatric Interview version Plus (MINI-PLUS).

### Assessments

#### Diagnostic criteria

The PMDD was assessed only in the second wave. The diagnosis of PMDD was assessed using the MINI-PLUS by trained psychologists. MINI-PLUS is a short structured interview based on the DSM-IV, validated in Brazilian Portuguese.^14^

#### Outcome

Suicide risk is the outcome of the present study, and it was also evaluated through the MINI-PLUS interview. The suicide risk evaluation includes six questions that assess suicidal intention, planning and previous attempts. Subjects who answer yes to any of the six questions are classified as having current suicide risk,^14^ according to the classification proposed by the MINI-PLUS interview. We are also presenting a description of the severity of suicide risk among individuals presenting suicide risk, by using the cut-offs suggested by the MINI-PLUS interview: low suicide risk (score range from 1 to 5), moderate suicide risk (score range from 6 to 9), and high suicide risk (score ≥ 10).^14^

#### Independent variables

The subjects answered a self-reported questionnaire to provide sociodemographic information that included the following information: age, skin color, current occupation, and marital status. The socioeconomic status was assessed through the Brazilian Association of Research Companies classification,^15^ which is based on the total of material goods and the householder's schooling. In this study, "A+B" refers to the highest economic classification, "C" to the middle class, and "D+E" to the lowest economic classification. Furthermore, other psychiatric disorders were also evaluated using the MINI-PLUS interview (major depressive disorder [MDD], bipolar disorder, current panic disorder, current agoraphobia, current social phobia, current obsessive compulsive disorder, current generalized anxiety disorder [GAD], and current posttraumatic stress disorder), and substance abuse or dependence was assessed through the Alcohol, Smoking, and Substance Involvement Screening Test (ASSIST).^16^ The ASSIST screening test evaluated the current use (last 3 months) of the following substances: tobacco, alcohol, cannabis, cocaine, inhalants, sedatives, hallucinogens, opioids, and other drugs. The cut-off used was 4. For the purpose of the present study, we grouped together the illicit substances (cannabis, cocaine, inhalants, sedatives, hallucinogens, opioids, and other drugs), and alcohol and tobacco were described separately.

The severity of depressive symptoms was evaluated by the Montgomery-Åsberg Depression Rating Scale (MADRS).^17^ The biological rhythms were evaluated through the Biological Rhythm Assessment Interview in Neuropsychiatry (BRIAN). This scale assesses the biological rhythms disruption in four domains: sleep, activities, social rhythm, and eating patterns.^18^ Childhood trauma was assessed using the Childhood Trauma Questionnaire (CTQ). This instrument investigates five traumatic components: physical abuse, emotional abuse, sexual abuse, physical neglect, and emotional neglect.^19^ The Short Functional Assessment Test (FAST) was used to assess the overall functioning. There are 24 items that assess impairment or disability in six specific areas of functioning: autonomy, occupational functioning, cognitive functioning, financial issues, interpersonal relationships, and leisure.^20^

### Statistical analysis

Data entry was performed using the Open Data Kit (ODK) software and then the data was analyzed using the Statistical Package for the Social Sciences (SPSS) version 22. The distribution of the numeric variables was tested using the Shapiro-Wilk test. All continuous data presented a non-normal distribution. Thus, continuous data were tested using the Mann-Whitney *U* test and presented by median and interquartile range. The comparison between two categorical variables were tested using the chi-square test. Finally, we conducted a multivariate analysis using logistic regression. In this multivariate model, we included suicide risk as our outcome, and all independent variables potentially associated with the outcome (p < 0.200) were included as the predictors in the model. A final model was reached using a manual stepwise removal of each non-statistically significant variable.

### Ethical considerations

The larger study was approved by the research ethics committee of the Universidade Católica de Pelotas (UCPel) under protocol number 2008/118. All participants signed a written informed consent form before their participation in the study.

## Results

The prevalence of suicide risk in women with PMDD was 28.1% (n = 36). Out of the 36 individuals with suicide risk, the majority was presenting low suicide risk (n = 28, 77.8%), and 22.2% (n = 8) were presenting moderate or high suicide risk. Table 1 shows the sociodemographic and clinical characteristics between women with PMDD with and without suicide risk. In the crude analysis, the prevalence of suicide risk was greater among women with a non-white skin color (p = 0.026), who belonged to lower socioeconomic class (p = 0.011), with diagnosis of MDD (p = 0.033), bipolar disorder (p = 0.032), current panic disorder (p = 0.008), current agoraphobia (p = 0.008), and tobacco abuse/dependence (p = 0.049).

**Table 1:** Sociodemographic and clinical factors associated with suicide risk in women with premenstrual dysphoric disorder (PMDD)

		Suicide risk		p-value
		No (n = 92)	Yes (n = 36)	
Age		27.00 (24.00-28.00)	27.00 (24.00-28.00)	0.751
			
		**n (%)**	**n (%)**	
Current occupation				0.580
	No	31 (33.7)	14 (38.9)	
	Yes	61 (66.3)	22 (61.1)	
			
Skin color				**0.026**
	White	63 (68.5)	17 (47.2)	
	Non white	29 (31.5)	19 (52.8)	
			
Has a partner				0.400
	No	30 (32.6)	9 (2.0)	
	Yes	62 (67.4)	27 (75.0)	
			
Socioeconomic class				**0.011**
	Upper (A/B)	44 (48.4)	8 (22.2)	
	C	43 (47.3)	23 (63.9)	
	Lower (D/E)	4 (4.4)	5 (13.9)	
			
Major depressive disorder				**0.033**
	No	55 (59.8)	14 (38.9)	
	Yes	37 (40.2)	22 (61.1)	
			
Bipolar disorder				**0.032**
	No	79 (85.9)	25 (69.4)	
	Yes	13 (14.1)	11 (30.6)	
			
Current panic disorder				**0.034**
	No	91 (98.9)	32 (88.9)	
	Yes	1 (1.1)	4 (11.1)	
			
Current agoraphobia				**0.008**
	No	80 (87.0)	24 (66.7)	
	Yes	12 (13.0)	12 (33.3)	
			
Current social phobia				0.127
	No	85 (92.4)	30 (83.3)	
	Yes	7 (7.6)	6 (16.7)	
			
Current obsessive compulsive disorder				0.267
	No	90 (97.8)	33 (91.7)	
	Yes	2 (2.2)	3 (8.3)	
			
Current generalized anxiety disorder				0.068
	No	81 (88.0)	27 (75.0)	
	Yes	11 (12.0)	9 (25.0)	
			
Tobacco abuse/dependence				**0.049**
	No	74 (80.4)	23 (63.9)	
	Yes	18 (19.6)	13 (36.1)	
			
Alcohol abuse/dependence				0.085
	No	68 (73.9)	21 (58.3)	
	Yes	24 (26.1)	15 (41.7)	
			
Illicit substance abuse/dependence				0.137
	No	83 (90.2)	29 (80.6)	
	Yes	9 (9.8)	7 (19.4)	

Bold type denotes statistical significance.

**Figure 1:**
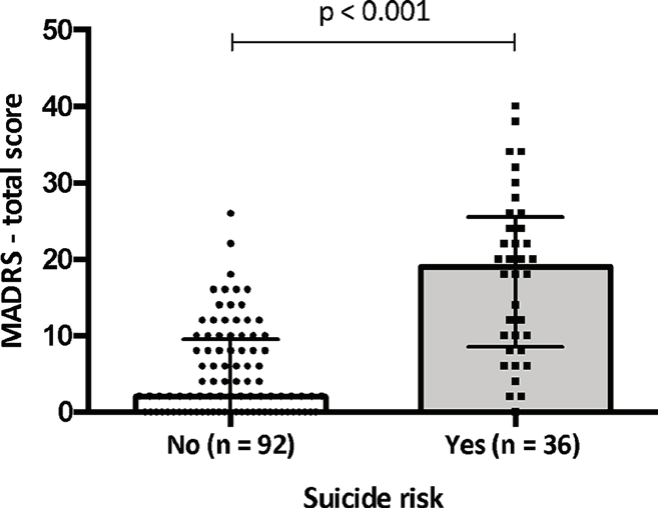
Severity of depressive symptoms and suicide risk in women with PMDD. MADRS = Montgomery-Åsberg Depression Rating Scale; PMDD = premenstrual dysphoric disorder.

Figure 1 depicts the association between severity of depressive symptoms and suicide risk in women with PMDD. Women with PMDD and suicide risk showed a greater severity of depressive symptoms (19.00 [IQR: 8.50-25.50]) when compared to women with PMDD without suicide risk (2.00 [IQR: 0.00-9.50], p < 0.001).

**Figure 2:**
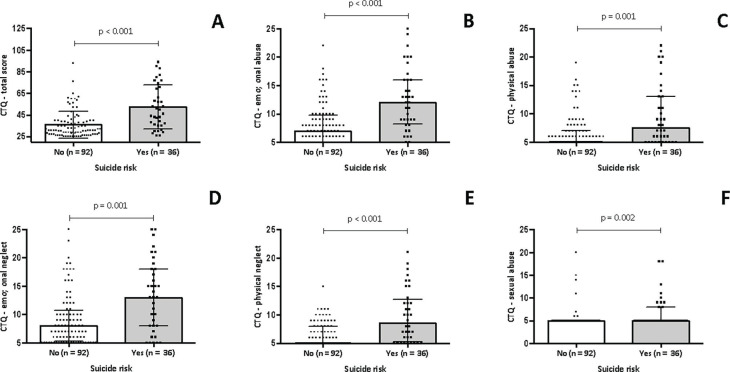
Childhood trauma and suicide risk in women with PMDD. CTQ = Childhood Trauma Questionnaire; PMDD = premenstrual dysphoric disorder.

Figure 2 shows the association between childhood trauma and suicide risk in women with PMDD. Figure 2A shows that individuals with PMDD and suicide risk have a greater frequency of traumatic experiences (48.00 [IQR: 35.25-70.50]) when compared to individuals with PMDD without suicide risk (31.00 [IQR: 27.00-39.75], p < 0.001). Figure 2B shows that individuals with PMDD and suicide risk had greater occurrence of emotional abuse (12.00 [IQR: 8.25-16.00]) when compared to those with PMDD without suicide risk (7.00 [IQR: 5.00-9.75], p < 0.001). Figure 2C shows that individuals with PMDD and suicide risk had greater scores in the physical abuse domain (7.50 [IQR: 5.00-13.00]) when compared to those with PMDD without suicide risk (5.00 [IQR: 5.00-7.00], p = 0.001). Figure 2D shows that individuals with PMDD and suicide risk had greater scores on the emotional neglect domain (13.00 [IQR: 8.00-18.00]) when compared to those with PMDD without suicide risk (8.00 [IQR: 5.25-10.75], p = 0.001). Figure 2E shows that individuals with PMDD and suicide risk had higher scores in the physical neglect domain (8.50 [IQR: 5.25-12.75]) when compared to those with PMDD without suicide risk (5.00 [IQR: 5.00-8.00], p < 0.001). Lastly, Figure 2F shows that individuals with PMDD and suicide risk had greater scores on the sexual abuse domain (5.00 [IQR: 5.00-8.00]) when compared to those with PMDD without suicide risk (5.00 [IQR: 5.00-5.00], p = 0.002).

**Figure 3:**
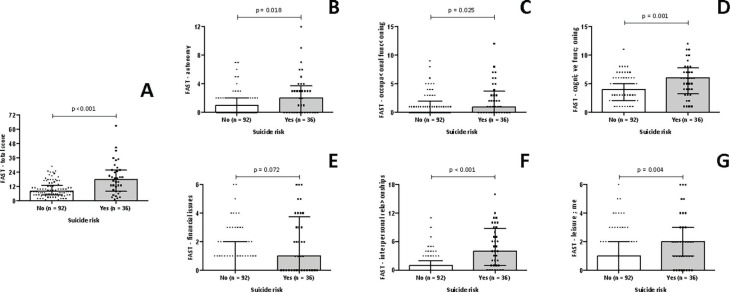
Functioning and suicide risk among women with PMDD. FAST = Short Functional Assessment Test; PMDD = premenstrual dysphoric disorder.

Figure 3 depicts the association between functioning and suicide risk among women with PMDD. Figure 3A shows that women with PMDD and suicide risk showed greater functional impairment (18.00 [IQR: 8.25-26.00]) when compared to those with PMDD without suicide risk (8.00 [IQR: 5.00-13.00], p < 0.001). According to Figure 3B, women with PMDD and suicide risk showed greater impairment in autonomy (2.00 [IQR: 0.00-3.75]) when compared to women with PMDD without suicide risk (1.00 [IQR: 0.00-2.00], p = 0.018), and Figure 3C shows that women with PMDD and suicide risk had greater occupational functioning impairment (1.00 [IQR: 0.00-3.75]) when compared to women with PMDD without suicide risk (0.00 [IQR: 0.00-2.00], p = 0.025). Figure 3D showed that women with PMDD and suicide risk showed greater cognitive functioning impairment (6.00 [IQR: 3.25-7.75]) when compared to those with PMDD without suicide such risk (4.00 (IQR: 2.00-5.00), p = 0.001), and Figure 3F showed that women with PMDD and suicide risk had greater impairment in interpersonal relationships (4.00 [IQR: 1.00-8.75]) when compared to women with PMDD without suicide risk (1.00 [IQR: 0.00-2.00], p < 0.001). Figure 3G showed that woman with PMDD and suicide risk showed greater impairment in leisure time (2.00 [IQR: 1.00-3.00]) when compared to those with PMDD without suicide risk (1.00 [IQR: 0.00-2.00], p = 0.004). Lastly, Figure 3E shows that there is no difference between groups regarding the financial issues domain.

**Figure 4:**
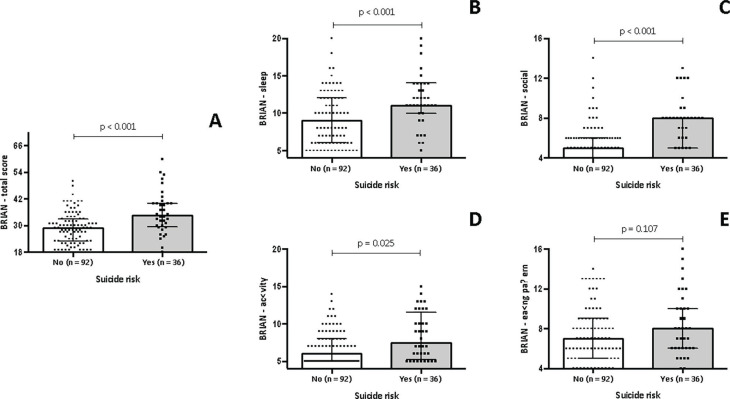
Biological rhythms and suicide risk in women with PMDD. BRIAN = Biological Rhythm Assessment Interview in Neuropsychiatry; PMDD = premenstrual dysphoric disorder.

Figure 4 shows the association between biological rhythms and suicide risk in women with PMDD. Figure 4A shows that women with PMDD and suicide risk had greater biological rhythms disruption (34.50 [IQR: 29.25-40.00]) when compared to women with PMDD without suicide risk (29.00 [IQR: 23.00-33.00], p < 0.001). Figure 4B shows that women with PMDD and suicide risk had greater disruption in the sleep domain (11.00 [IQR: 10.00-14.00]) when compared to those with PMDD without suicide risk (9.00 [IQR: 6.00-12.00], p < 0.001). According to Figure 4C, women with PMDD and suicide risk also showed greater disruption in the social domain (8.00 [IQR: 5.00-8.00]) when compared to those with PMDD without suicide risk (5.00 [IQR: 4.00-6.00], p < 0.001). Figure 4D shows that women with the PMDD and suicide risk have greater impairment in the activity domain (7.50 [IQR: 5.25-11.50]) when compared to those with PMDD without suicide risk (6.00 [IQR: 5.00-8.00], p = 0.025). Finally, Figure 4E shows that there is no difference between groups regarding the eating pattern domain.

The final logistic regression model is presented on Table 2. This model showed that the variables that remained statistically significantly associated with suicide risk in women with PMDD were: non-white skin color (p = 0.018), presence of current panic disorder (p = 0.048), a greater score on the childhood trauma (p = 0.010), and a greater severity of depressive symptoms (p < 0.001).

**Table 2:** Multivariate analysis for the factors associated with suicide risk in women with premenstrual dysphoric disorder (PMDD)

Variable		OR (95%CI)	p-value
Skin color			0.018
	White	1	
	Non-white	4.18 (1.28-13.61)	
		
Current panic disorder			0.048
	No	1	
	Yes	18.71 (1.02-343.27)	
		
CTQ – total score		1.04 (1.01-1.08)	0.010
MADRS – total score		1.22 (1.12-1.32)	< 0.001

95%CI = 95% confidence interval; CTQ = Childhood Trauma Questionnaire;

MADRS: Montgomery-Åsberg Depression Rating Scale; OR = odds ratio.

## Discussion

Our population-based study showed that 28% of young women with PMDD presented with suicide risk. The main factors associated with suicide risk in this sample were non-white skin color, greater severity of depressive symptoms, current panic disorder, and greater lifetime childhood trauma. Our findings are consistent with previous research indicating that non-white race, presence of psychiatric comorbidity, and childhood trauma are factors linked with suicidality.^21,22^ For instance, a recent study by Pilver et al. reported that women with PMDD were significantly more likely than women with no premenstrual symptoms to endorse suicidal ideation, plans, and attempts, and when PMDD status was accounted for, race/ethnicity was still significantly associated with suicidal ideation.^21^ Even though we did not assess race specifically in our study, the characteristic of skin color likely reflects differences in race and ethnicity, and thus, our finding that non-white skin color was a factor associated with suicide risk in PMDD appears to be in alignment with Pilver et al. In addition, it has been suggested that skin color may represent a distal social risk factor for understanding suicide risk.^23^ Future research should aim to better understand the role that race-related factors and systemic issues play in the development and presentation of PMDD and their impact on suicide risk, so that culturally-congruent interventions may be devised.^10^

Previous studies have also shown that the relationship between suicidality and PMDD is significant even after controlling for major depression and other psychiatric disorders.^4,9,21,24^ Pilver et al. proposed that though MDD and PMDD have shared variance, PMDD has an independent effect on suicidality. In their study, they found that the association between PMDD and non-fatal suicidal behaviors was comparable to that of MDD and non-fatal suicidal behaviors, which sheds some light into why depression may add risk for suicidality among individuals with PMDD, as it is a distinct disorder accompanied by suicide risk of its own.^21^ In our multivariate model, although MDD comorbidity did not emerge as a statistically significant predictor of suicide risk in young adults with PMDD, the severity of depressive symptoms did. These results suggest that the severity of current depressive symptoms may be more relevant than a lifetime history of MDD when predicting suicide risk in women with PMDD. Indeed, Wikman et al. found that among a sample of women with PMDD, those who reported current suicidal ideation also endorsed higher overall depressive symptomatology during the late luteal phase, compared to subjects with no suicidal ideation; a greater level of depressive symptoms was positively associated with current suicidal ideation.^25^ Our findings align with these results and highlight the importance of assessing severity of current depressive symptoms over and above comorbidity alone when treating PMDD populations.

Regarding anxiety, we found that the prevalence of current panic disorder was greater in women with PMDD and suicide risk than among women with PMDD without suicide risk. Prior studies have shown that depressive symptoms and anxiety are higher among women with PMDD compared to controls,^26^ and some studies suggest that there may be a shared biological basis between PMDD and panic disorder specifically. Challenge studies, which involve a provocation paradigm, have been found to induce panic in both individuals with panic disorder and those with PMDD.^27,28^ Moreover, a study by Baca-Garcia et al. reported that among a sample of female suicide attempters, attempters with PMDD had higher rates of panic disorder (10%) with agoraphobia compared to attempters without PMDD (2%). This result almost reached significance (p = 0.07) and supports our conclusion that panic disorder may be an important risk factor for suicidality in PMDD. Interestingly, one study by Yen et al. found that women with GAD had greater levels of luteal anxiety than those with PMDD but no GAD.^29^ As higher rates of completed suicide are typically observed in the luteal phase,^9^ anxiety may exacerbate the affective component of PMDD during the luteal phase. Further research may be valuable in delineating which symptoms and clinical profiles of anxiety, if any, are associated with suicidality in PMDD. This may facilitate deeper understanding of which individuals should be selected for further screening and intervention.

Our results also reveal childhood trauma as a factor associated with suicide risk among women with PMDD. Prior research has suggested that childhood trauma increases the risk of suicide attempt by 15-fold,^30^ and a Turkish study by Soydas et al. found that those with PMDD were more likely to report higher overall childhood trauma scores, particularly emotional abuse, emotional neglect, physical abuse, and sexual abuse. It may be that the presence of childhood trauma, compounded with the affective symptoms of PMDD, collectively contributes to high stress and emotional disturbance. This may be further exacerbated by the presence of comorbid conditions, such as depression and anxiety, conferring suicide risk in PMDD. Unfortunately, individuals with PMDD may be disadvantaged by poor social connectedness^31^ and impaired ability to engage in other protective behaviors that could potentially buffer the suicide risk resultant from trauma and/or comorbid psychiatric conditions.

Our results hold clinical relevance, as greater understanding of risk factors associated with suicide risk in PMDD may enable clinicians to identify at-risk individuals who could benefit from further screening and intervention, especially in young adults. Notably, individuals with PMS or PMDD who attempt suicide have been found to have higher levels of impulsivity and aggressiveness compared to those without PMS or PMDD who attempt suicide.^6^ Further investigations into suicide risk in women with PMDD may drive the development of interventions and preventative strategies tailored for this disorder. Though suicidal ideation and behavior are not PMDD-specific, the cyclical nature of menstruation as well as the specific population affected are unique features of the condition that should be held in mind when developing treatment and interventional plans.

Our findings should be interpreted considering some study limitations. First, although the diagnosis of PMDD was assessed by trained psychologists through a clinical interview based on DSM-IV criteria, the daily prospective charting for a minimum of 2 months was not performed. Second, it is known that predictors for suicide ideation might differ from predictors for suicide attempt, and classifying suicide risk using a dichotomous variable to may be adding heterogeneity to our outcome. However, we analyzed suicide ideation and attempt together and did not stratify the sample by the severity of suicide risk, due to our limited sample size of individuals presenting with suicide risk. Third, the phase of the menstrual cycle was not assessed in this study. Finally, due to our study design, we cannot infer causality. Despite these limitations, we included a large sample recruited from the community. In addition, our study assessed the factors associated with suicide risk in a sample of young adult women with PMDD, which is a highly vulnerable population to present suicide risk. Finally, assessing the factors associated with suicide risk in youth with PMDD may contribute to the early detection of suicide risk in this population, as well as to the development and implementation of preventive strategies.

## Conclusion

In conclusion, we showed that 28% of young women with PMDD exhibit suicide risk. Non-white skin color, current panic disorder, history of childhood trauma, and greater severity of current depressive symptoms were associated with suicide risk in this population. These results may inform strategies for early identification and tailored interventions for those women with PMDD who are vulnerable to suicidality.

## Data Availability

The data that support this study are available from the authors upon request.
